# Development of novel transgene‐free high‐oleic peanuts through CRISPR‐Cas9‐mediated gene editing of two *AhFAD2* homologues

**DOI:** 10.1111/pbi.70256

**Published:** 2025-07-13

**Authors:** Lei Shi, Huanhuan Zhao, Han Liu, Xiaobo Wang, Xiaona Li, Pengyu Qu, Lulu Xue, Bingyan Huang, Feiyan Qi, Xiaodong Dai, Jing Xu, Wenzhao Dong, Xinyou Zhang

**Affiliations:** ^1^ Institute of Crop Molecular Breeding Henan Academy of Agricultural Sciences/Key Laboratory of Oil Crops in Huang‐Huai‐Hai Plains, Ministry of Agriculture/Henan Provincial Key Laboratory for Oil Crops Improvement/National and Provincial Joint Engineering Laboratory for Peanut Genetic Improvement/The Shennong Laboratory/National Invocation Center for Bio‐Breeding Industry/Henan Biological Breeding Center Co., Ltd. Zhengzhou China; ^2^ School of Agricultural Sciences Zhengzhou University Zhengzhou China; ^3^ College of Life Sciences Zhengzhou University Zhengzhou China

**Keywords:** peanut (*Arachis hypogaea* L.), CRISPR/Cas9, genome editing, *FAD2*, high‐oleic

Cultivated peanut (*Arachis hypogaea* L.), an allotetraploid crop (2*n* = 4*X* = 40, AABB), is a vital oilseed crop cultivated across temperate and tropical zones in over 100 countries (Norden *et al*., 1987). Commodity peanut seeds typically comprise 45%–56% oil, with two major fatty acids, oleic acid (C18:1) at approximately 42% and linoleic acid (C18:2) at about 37%. Oleic acid is notably more oxidatively stable than linoleic acid, offering an extended shelf life (Martín *et al*., 2018), preventing the development of off‐flavours (Barkley *et al*., 2013) and reducing the formation of nutritionally undesirable trans fatty acids (Chu *et al*., 2007).

Fatty acid desaturase 2 (*FAD2*) catalyses the conversion of oleic acid to linoleic acid by introducing a second double bond to oleic acid within the endoplasmic reticulum (ER) (Okuley *et al*., 1994). Genetic studies have shown that the simultaneous loss of function in two homeologous genes, *AhFAD2A* and *AhFAD2B*, located on chromosome 9 of the A subgenome and chromosome 19 of the B subgenome, respectively, in *A. hypogaea*, results in a high‐oleic acid trait in peanut, characterized by approximately 80% oleic acid and just 2% linoleic acid in peanut oil.

China is the world's largest peanut producer and consumer of peanuts, with peanut production ranking first among oilseed crops in the country. Despite the dominance of the upright peanut varieties in China, no high‐oleic mutant or germplasm has been identified originally in the country to date. In this study, we leveraged CRISPR‐Cas9 technology to knockout *AhFAD2A* and *AhFAD2B* genes, resulting in the generation of transgene‐free high‐oleic peanut plants.

In our experiment, the expression of Cas9 was driven by the 2× CaMV35S promoter, while the Arabidopsis AtU6 promoter directed sgRNA expression (Figure 1a). The sgRNAs targeted regions adjacent to and downstream of the start ATG codon of *AhFAD2A* and *AhFAD2B* (Figure 1b). The CRISPR construct was introduced into the elite peanut cultivars Yuhua9326 via microprojectile bombardment of embryogenic tissue cultures. Following three consecutive cultures of 3 weeks each on MS medium containing hygromycin (Hyg), 13 independent T_0_ Hyg‐resistant embryogenic tissue lines were obtained. DNA from the Hyg‐resistant calli was extracted and amplified using gene‐specific primers (Table S1) targeting *AhFAD2A*, *AhFAD2B* and a pseudogene. Sanger sequencing of the amplicons revealed that 11 out of 13 lines (84.6%) exhibited mutations in two or all targeted sequences (Figure 1c). Within the editing window, seven out of 39 target sites displayed a single peak on the chromatogram, indicating homozygous mutations, while 25 displayed overlapping peaks, signifying heterozygous mutations with two distinct alleles. Seven target sites retained a single peak corresponding to the unedited wild‐type (WT) allele. Notably, one line was homozygous for mutations in both *AhFAD2A* and *AhFAD2B*. Overall, the editing efficiency for *AhFAD2A*, *AhFAD2B* and the pseudogene was approximately 82.0% (32/39). Diverse mutation types were observed at on‐target sites, including deletions and insertions of varying lengths. These included single‐nucleotide changes (e.g. T), short sequences (e.g. TT, TC and TGA), as well as longer insertions or deletions (InDels) ranging from 61 to 597 bp (Figure S1). All observed non‐multiple‐of‐3‐bp mutations resulted in the effective knockout of the *AhFAD2A*/*B* genes, demonstrating the precision and versatility of the CRISPR‐Cas9 system in peanut gene editing.

**Figure 1:**
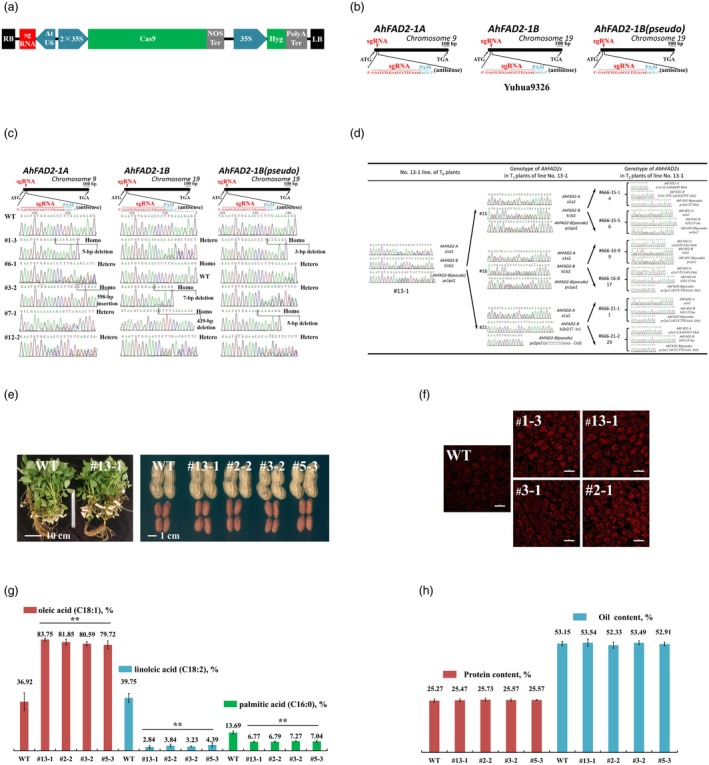
Development of a novel transgene‐free high‐oleic peanut via CRISPR‐Cas9‐mediated gene editing. (a) Schematic diagram of the T‐DNA region, illustrating the CRISPR‐Cas9 construct used for gene editing of two *AhFAD2* homologues. (b) Schematic representation of sgRNA target sites for *AhFAD2A* and *AhFAD2B*. The sgRNA sequences are highlighted in red, and PAM sequences are marked in blue. (c) Chromatograms of Sanger sequencing results for the sgRNA sites in *FAD2A*, *FAD2B* and pseudogene in T_0_ mutants. WT, wild‐type (Yuhua9326); homo, homozygous mutations; hetero, heterozygous mutation. (d) Genetic transmission analysis of CRISPR‐Cas9‐edited *AhFAD2A*, *AhFAD2B* and the pseudogene across T_0_–T_2_ generation. (e) Phenotypic comparison of WT and CRISPR‐Cas9 gene‐edited lines, highlighting no observable morphological differences. (f) A confocal section of seed from WT and gene‐edited plants, stained with Nile red. Scale bars = 100 μm. (g, h) Comparative analysis of oleic acid, linoleic acid, palmitic acid, protein and oil content between WT and four independent T_3_ lines. Fatty acid composition was quantified using GC–MS, while protein and oil content were measured using the Kjeldahl method and Soxhlet extraction, respectively. Samples were collected from 3 to 6 plants per line. ** indicates significant differences between WT and edited lines at the 0.01 probability level.

Out of the 11 gene‐edited T_0_ lines, eight successfully produced seeds in varying quantities following regeneration and grafting. While the T_0_ plants carried heterozygous mutations at the targeted sites, their T_1_, T_2_ and T_3_ progeny exhibited both homozygous and heterozygous mutations. This observation suggests that the heteroduplexes resulted from the hybridization of distinct CRISPR‐induced mutations. In the edited plants analysed, the T_2_ progeny derived from different T_1_ plants retained consistent mutation profiles. For instance, the mutation types observed in line #13‐1 remained stable across its T_1_ and T_2_ offsprings (Figure 1d). This stability demonstrates that edited alleles introduced in the T_0_ plants can be reliably transmitted to subsequent generations, highlighting the heritability and precision of the CRISPR‐Cas9 gene‐editing system in peanuts.

A total of 15 T_3_ plants, derived from six CRISPR‐Cas9‐edited *AhFAD2A*/*B* mutant lines (lines #1‐3, #2‐1, #3‐1, #10‐1, #13‐1 and #20‐1), were analysed using MGI2000 whole‐genome sequencing at a 20× sequencing depth (Michno *et al*., 2020). To evaluate potential off‐target effects of CRISPR‐Cas9 in peanut plants, single nucleotide polymorphisms (SNPs) and InDels were detected in the edited plants and the WT Yuhua9326 plant using the reference genome of cultivar Yuanza9102 (reference genome link: https://doi.org/10.6084/m9.figshare.26551309.v1). No significant difference in the number of variations was observed between the edited lines and Yuhua9326. However, considerable genetic variation was noted between Yuhua9326 and reference genome sequences (Table S2). Across the 15 edited plants, 427–5836 unique SNPs and 529–3125 InDels were identified when compared to Yuhua9326 and the reference genome. Importantly, most variations lacked protospacer‐adjacent motifs (PAMs), suggesting that these genetic changes arose from somaclonal variation or inherent variation from maternal plants, rather than off‐target effects of CRISPR‐Cas9 editing (Table S2). To comprehensively evaluate off‐target mutations, the sgRNA and their PAM sequences were aligned with the reference genome using CRISPR‐P and Cas‐OFFinder software (Li *et al*., 2019). With ≤5 mismatches in the sgRNA and PAM sequences, 2128 (PAM: NGG), 1315 (PAM: NAG) and 1785 (PAM: NGA) potential off‐target sites were predicted. Whole‐genome sequencing (WGS) detected a very low off‐target mutation frequency, identifying only 18 InDels (PAM: NGG) across different CRISPR/Cas9 transgenic lines, none of which were located within the 10 most likely off‐target sites (Table S3). This indicates that no off‐target mutations were present in the sequenced edited lines, effectively ruling out off‐target effects as a concern. Among the 15 T_3_ offspring, six offspring derived from lines #1‐3, #13‐1 and #20‐1 were confirmed to have lost the transgenes, as verified by next‐generation sequencing (Table S4). Additionally, four T_4_ offspring derived from the non‐transgenic T_3_ plants of lines #1‐3, #13‐1 and #20‐1 were further analysed using WGS, and no transgenic elements were detected.

No morphological abnormalities were observed in the vegetative or reproductive growth of FAD2*A*/*B* knockout lines compared to the WT (Figure 1e). The fatty acid composition, oil content and protein content of seeds harvested from homozygous T_3_ plants with *AhFAD2A*/*B* double mutations were analysed using Nile red staining, gas chromatography, the Kjeldahl method and Soxhlet extraction. The results revealed a substantial increase in oleic acid content, rising from 36.92% in the WT to 83.75%, 81.85% and 80.59% in the edited lines, accompanied by a drastic reduction in linoleic acid content from 39.75% to 2.84%, 3.84% and 3.23%. Additionally, palmitic acid content was significantly reduced from 13.69% in the WT to 6.77%, 6.79% and 7.27% in the edited lines (Figure 1g). Notably, no significant differences were observed in protein or oil content between the knockout edited lines and the WT (Figure 1f,h).

In conclusion, we successfully generated high‐oleic peanut lines in the elite commercial cultivar Yuhua9326 by specifically disrupting the *AhFAD2A*/*B* genes. Moreover, we demonstrated that CRISPR‐Cas9 is a promising and efficient tool for precise and rapid improvement of elite peanut cultivars without negatively affecting growth or yield.

## Conflict of interest

The authors declare no conflict of interest.

## Author contributions

L.S. and X.Z. conceived the study; L.S., H.Z., H.L., X.W., X.L., P.Q., L.X., B.H., F.Q., X.D., J.X. and W.D. performed the experiments. L.S. wrote the manuscript and X.Z. revised the manuscript. All authors read and approved the final manuscript.

## Supporting information


Figure S1.
Table S1–S4.

## Data Availability

The raw sequencing data of *FAD2* gene‐edited plants generated in this study are available at Genome Sequence Archive (GSA, https://ngdc.cncb.ac.cn/gsa/) of the National Genomics Data Center, under submission accession number subCRA039668 associated with BioProject PRJCA038148.
