# Supplementary material for: Development of novel transgene‐free high‐oleic peanuts through CRISPR‐Cas9‐mediated gene editing of two AhFAD2 homologues
Source: Plant Biotechnol J. 2025 Jul 13;23(10):4618–20. doi: 10.1111/pbi.70256 (PMC12483977; doi:10.1111/pbi.70256)
Supplement: Supplementary file 1 — Figure S1. Table S1–S4. [file PBI-23-4618-s001.pdf]

Figure S1. Chromatograms depicting Sanger sequencing results of gene-edited alleles in *AhFAD2A*, *AhFAD2B*, and the pseudogene across T_0 to T_1 generation. The chromatograms illustrate various mutation types in the gene-edited alleles. Blue underlined nucleotides represent the residual sequences at the target sites. Red arrows mark the mutation position. Deleted sequences are highlighted within red rectangles, while inserted sequences and their corresponding chromatograms are enclosed within green lines.

Edited allele of *AhFAD2A* from #2-1-47 of the T_1 plants

(TTTC-deletion)

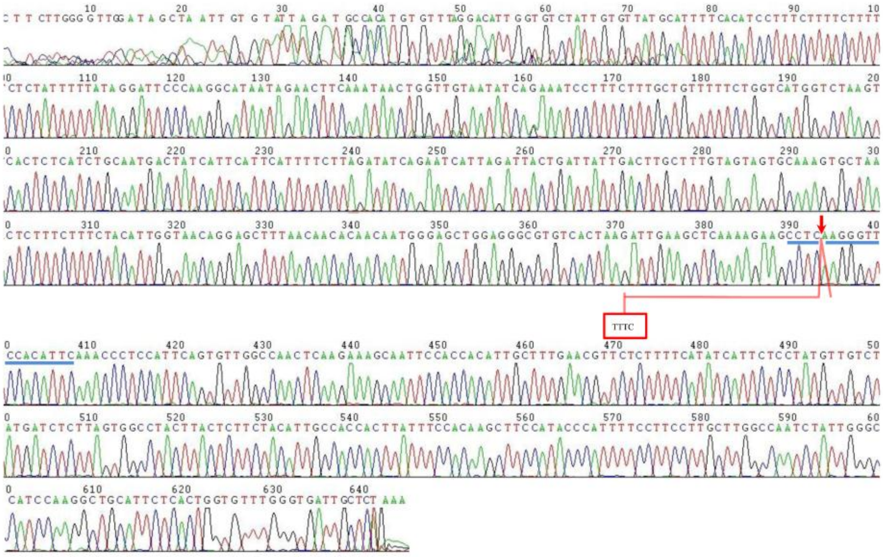

Edited allele of *AhFAD2A* from #20-1-10 of the T_1 plants

(TGGAACCCTTGAAAGAGGCTTCTTTTGAGCTTCAATCTTAGTG-deletion)

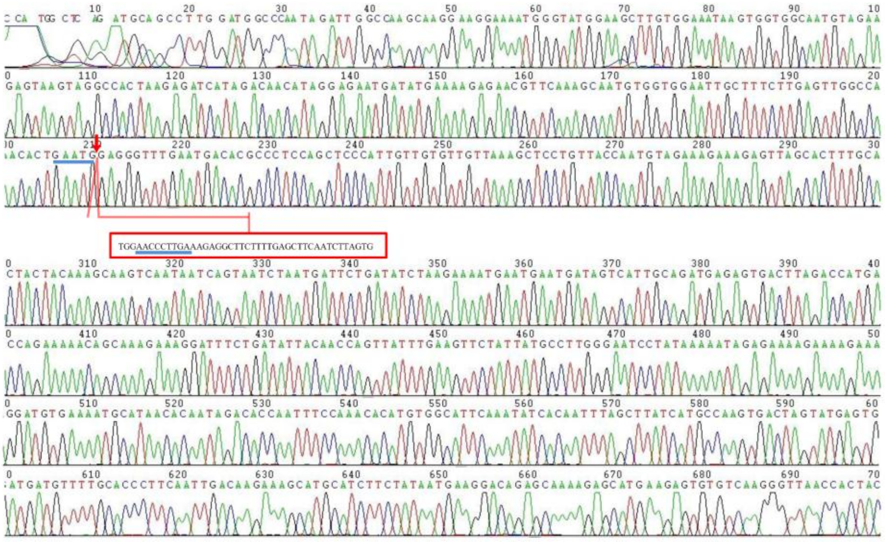

Edited allele of *AhFAD2A* from #1-3-B01(2) of the T_1 plants

(CAAGG-deletion)

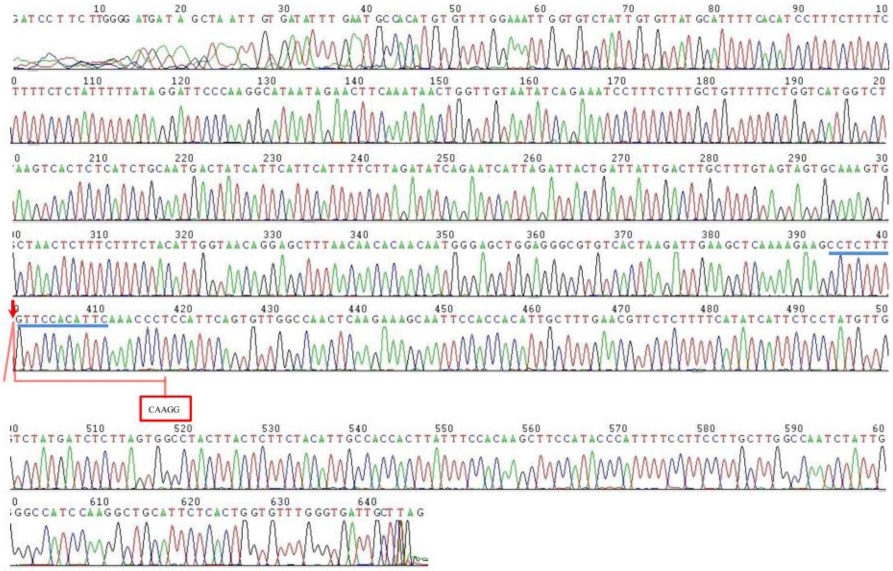

Edited allele of *AhFAD2A* from #1-2-14 of the T_1 plants

(CCTTG-deletion)

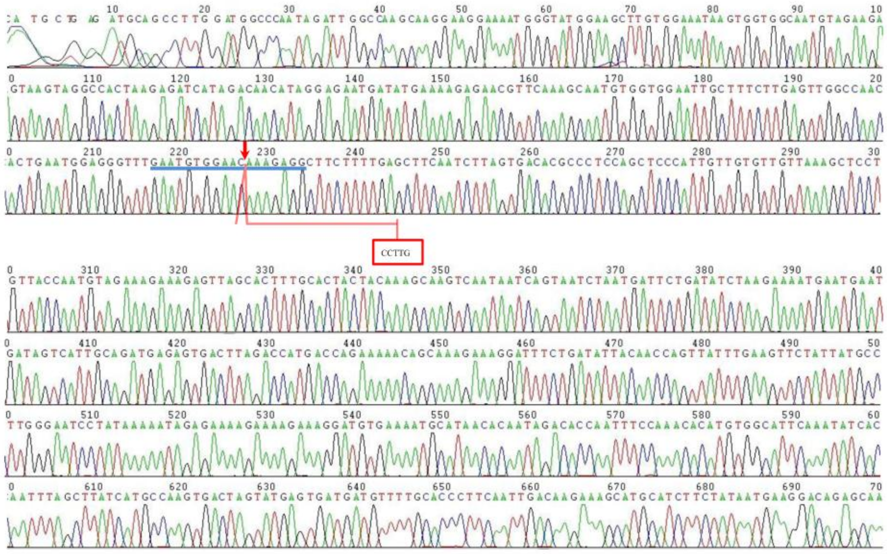

Edited allele of *AhFAD2A* from #1-3-19 of the T_1 plants

(AAGA-deletion)

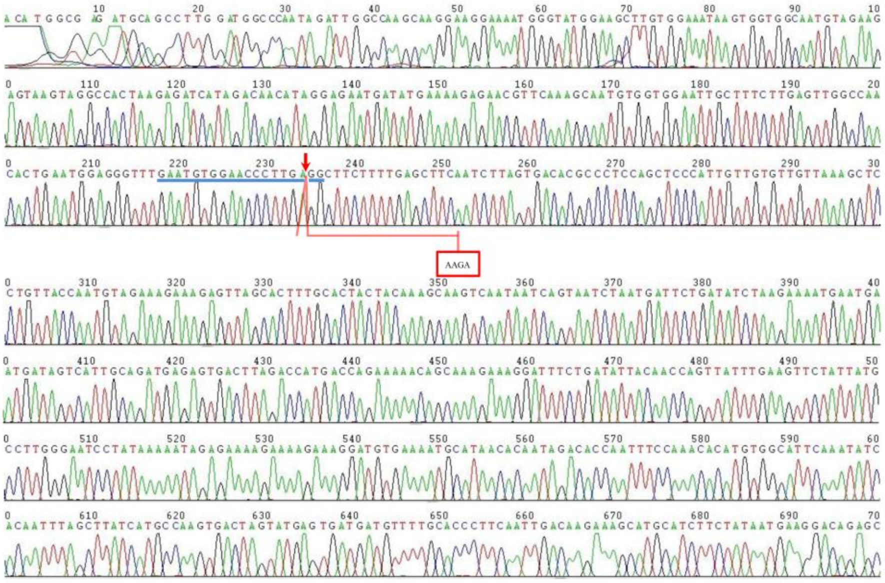

Edited allele of *AhFAD2A* from #2-2-13 of the T_1 plants (TTCTTCAAATTAAATTATAATTTGAGCTTTTCACATTCTCTCACACCAAAAATCTCTCATATGAGGAGTAGAAGTTCTAGAGATATAAATATATCATTTACTACTTATTTCTTTCTAGTATGAAAAGAGTCATCACAATATTGTATTAATATGTCTTTTCATTTCAAAATTGACTTTTTT-insertion)

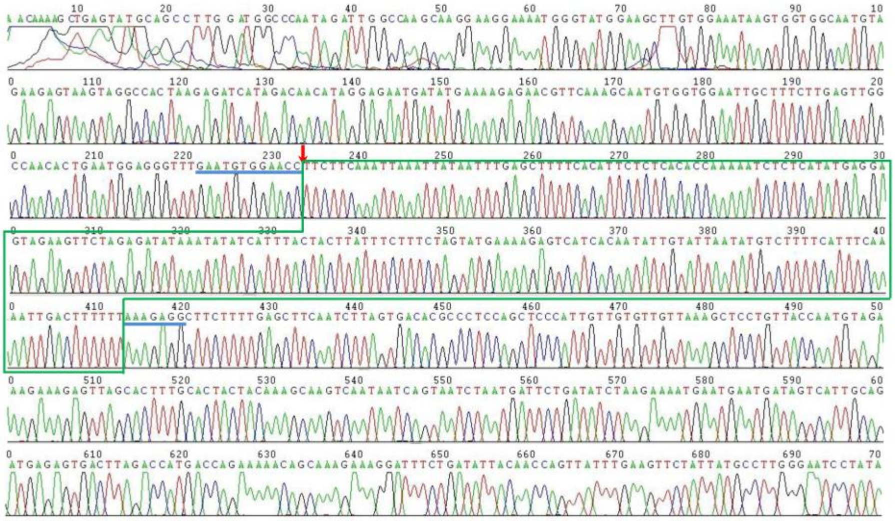

Edited allele of *AhFAD2A* from #5-3-5 of the T_1 plants

(AACCCTTGAAAGAG-deletion)

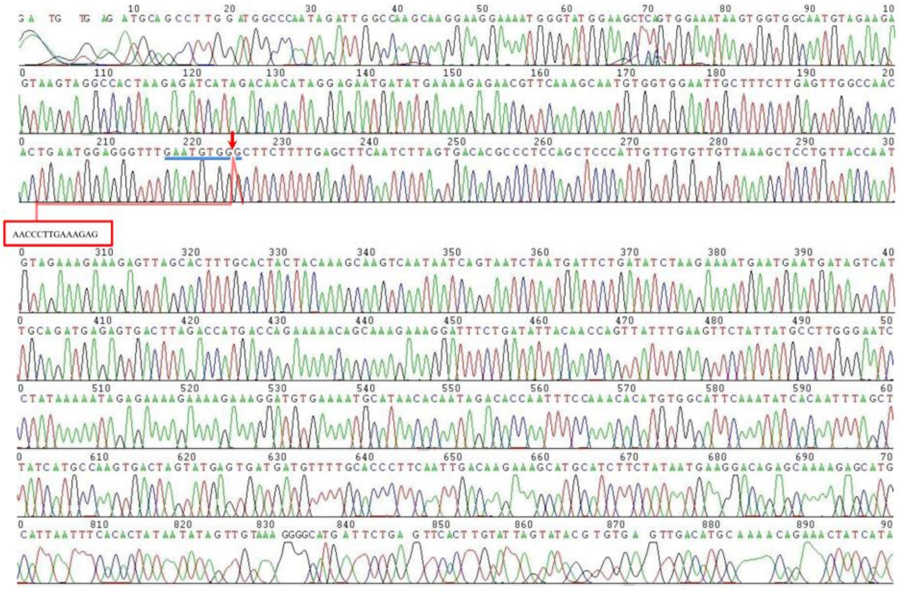

Edited allele of *AhFAD2B* from #7-1-10 of the T_0 plants (CCATTAAGAGATCATAGACAACATATGAGAATGATATGAAAAGAGAACGTTCAAAGCAATGTGGT GGAATTGCTTTCTTGAGTTGGCCAACACTGAATGGAGGGTTTGAATGTGGAACCCTTGAAAGAGG CTTCTTTTGAGCTTCAATCTTAGTGACACGCCCTCCAGCTCCCATTGTTGTGTTGTTAAAGCTCCTG TTACCAATGTAGAAAGAAAGAGTTAGCACTTTGCACTACTACAAAGCTAATGGTTCTGATATCTAA GAAAATGAATGAATGATAGTCATTGCAGATGAGAGTGACTTAAACCATGACCAGAAAAACAGCAA AGAAAGGATTTCTGATATTACAACCAGTTATTTGAAGTTCTATTATGCCTTGGGAATCCTATAAAAA TAGAGAAAAGAAAAGAAAGGATGTGAAAATGCCTAACACAATAGACA-deletion)

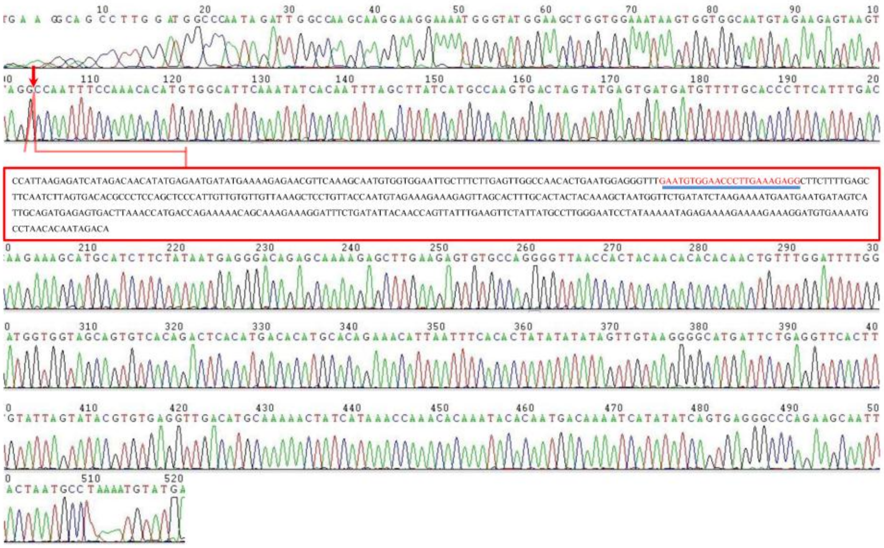

Edited allele of *AhFAD2B* from #13-1-17 of the T_1 plants

(T-insertion)

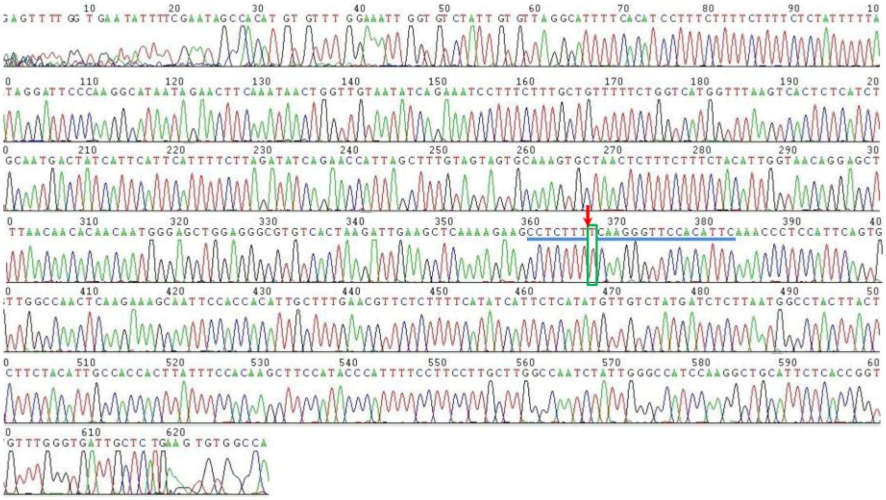

Edited allele of *AhFAD2B* from #13-1-18 of the T_1 plants

(TTCAAGGGTTC-deletion)

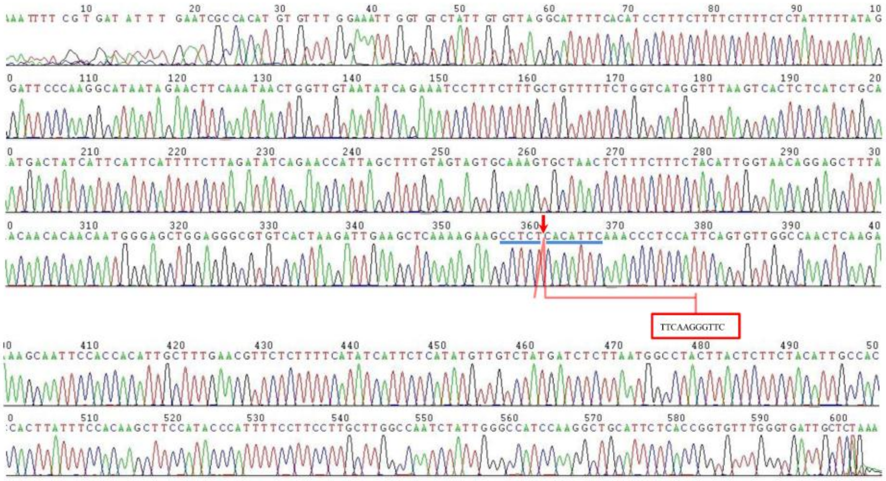

Edited allele of pseduo from #5-3-6 of the T_1 plants

(CCCTTGA -deletion)

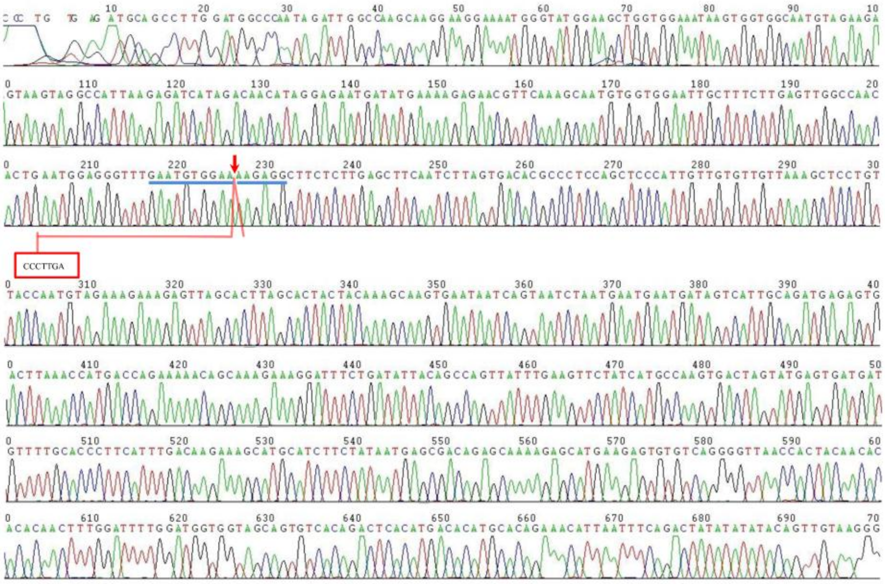

Edited allele of pseduo from #20-1-9 of the T_1 plants

(AAAGAGG -deletion)

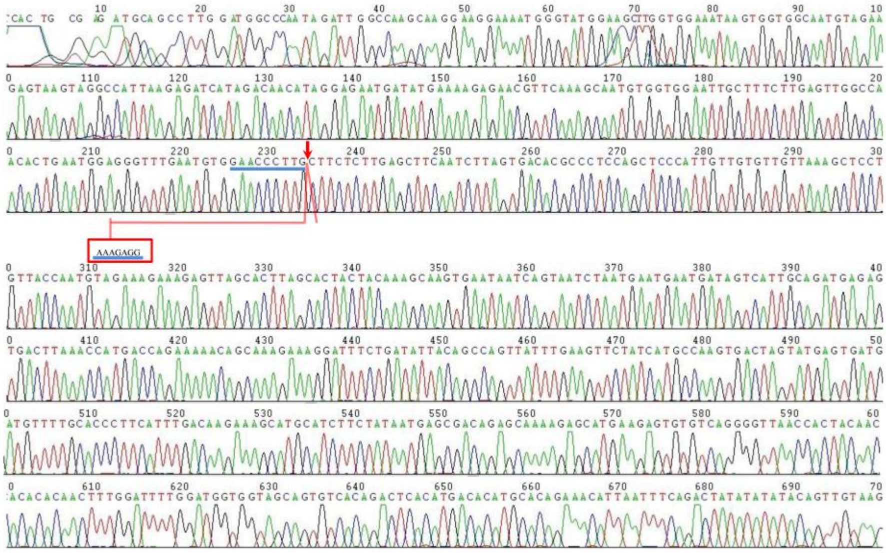

Edited allele of pseduo from #5-3-5 of the T_1 plants (GCCAACACTGAATGGAGGGTTTGAATGTGGAACCCTTGAAAGAGGCTTCTCTTG-deletion)

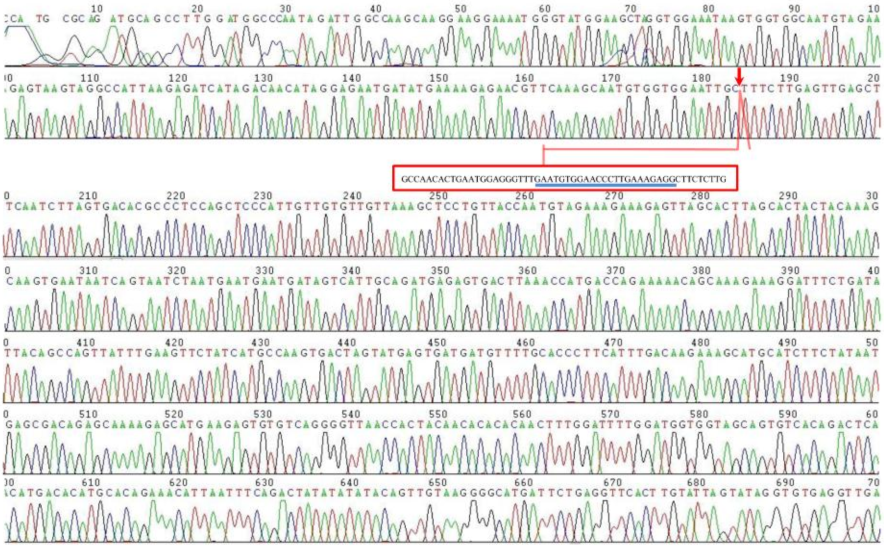

Edited allele of pseduo from #1-3-21 of the T_1 plants (GACGATTGACCGCGACGTGAAGGCCATC-insertion)

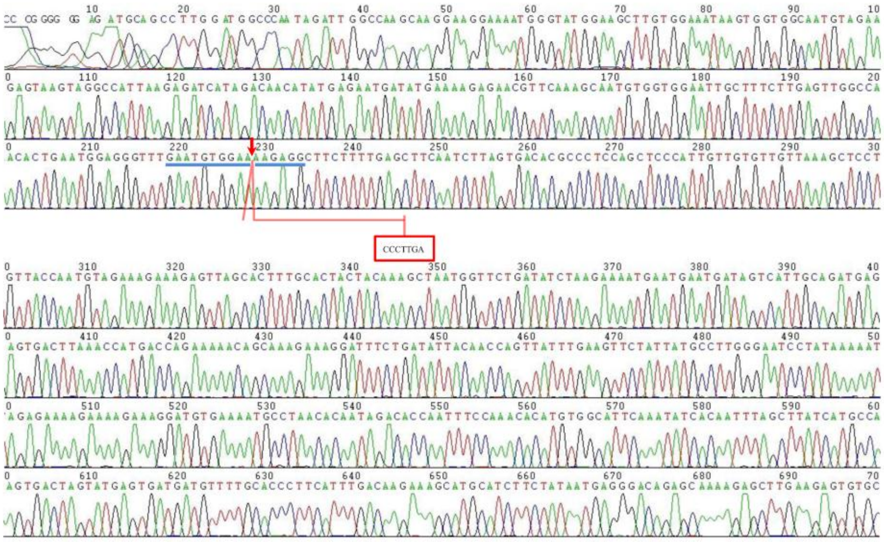

Edited allele of pseduo from #1-2-14 of the T_1 plants

(TGA -deletion)

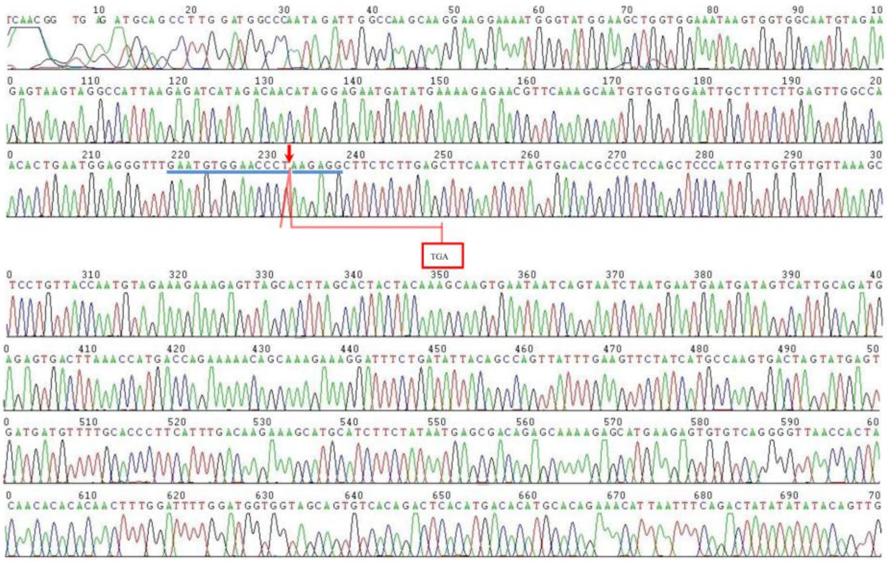

Edited allele of pseduo from #2-2-13 of the T_1 plants (GGAGCGAACGACCTACACCGAACTGAGATACCTACAGCGTGAGCTATGAGAAAGCGCCACGCTT CCCGAAGGGAGAAAGGCGACAAGCTCGAGTTTCTCCATAATAATGTGTGAGTAGTTCCCAGATAA GGGAATTAGGGAATAACATAATATCTGGATGAATTTAATTTCAAAATGCAAAGGTGATATATATTTAT TTATTATACCTTTAAATCGGCACCAGCACAATAGACACCAGGAATTGAATTGGTGATGATGGCAAC ATTAGTGGAAGAATCTTGATTAATCACCTCTAATGCATGCTTTAATTAACCCTCTCATTAATTTGGTG TCTATAACATTCTTATTTTTAGCCCTATCCAAAGTTACCTCGACAATCCCTACCCCAAAAAAAGAAT AAGT-insertion)

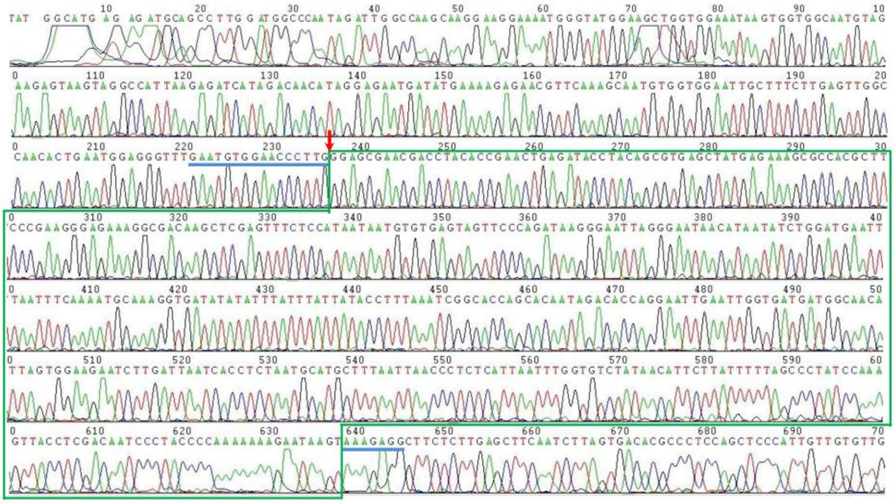

Table S 1 sequences of primers used in this study

	Purpose	Forward primer(5´–3´)	Reverse primer(5´–3´)
Identification of gene editing target sites	*AhFAD2A*	GGCATTAGTTTTGATGGCATG	
	AhFAD2B	TGCAAAACATCATCACTC	GGCCACACTCATGAGCAATC
	pseudogene	ATGTAGGTTAGCATTTGCCAAG	
	Sequencing for gene editing target regions of *AhFAD2A*, *AhFAD2B* and pseudogene	GGCCACACTCATGAGCAATC	

Table S2 The summary of total variations in wild type and CRISPR/Cas9-edited plants

Description plants	Lines vs Ref		Plants vs Ref/WT		Private variations	
	SNP	Indel	SNP	Indel	SNP	Indel
Yuhua9326 (WT)	493783	266735	\	\	\	\
#1-3-21-1-11	\	\	155953	93553	756	757
#1-3-2-3-30	\	\	157381	94219	540	910
#1-3-10-9-30	\	\	157878	98521	427	529
#1-3-10-5-30	\	\	159338	100017	641	676
#13-1-21-1-29	\	\	156392	95621	676	735
#1-3-10-7-11	\	\	161862	105602	1008	797
#13-1-16-8-17	\	\	161272	104443	1386	1031
#13-1-21-2-29	\	\	161874	105898	770	930
#1-3-11-1-30	\	\	155639	89017	1411	986
#3-1-26-2-33	\	\	167687	112224	5836	3125
#10-1-23-2-36	\	\	155886	93164	849	804
#10-1-23-1-39	\	\	158177	101747	835	721
#2-1-47-3-41	\	\	162144	105186	2909	1099
#13-1-16-5-46	\	\	157235	97188	1608	797
#13-1-15-1-4	\	\	152056	81530	1406	2057

Table S3 Identification of off-target mutations in Cas9-edited plants by whole genome sequencing

Cas9-edited plants/sgRNA	Cas9 mutations/No. of NGG sites (Ratio %)	Cas9 mutations/No. of NAG sites (Ratio %)	Cas9 mutations/No. of NGA sites (Ratio %)
FAD2	18 InDel /2128 (2.26)	0/1315 (0.00)	0/1785 (0.00)

Table S4 Statistics of the insertion sites of exogenous vector sequences in T_3 plants

Plants	#No. of insertions	Inserted Chromosome	Starting position	Ending position
#1-3-21-1-11	1	arahy.YZ9102.chr09	119761643	119762040
#1-3-10-7-11	1	arahy.YZ9102.chr09	119761642	119762042
#13-1-21-1-N5	8	arahy.YZ9102.chr12	5021984	5021985
		arahy.YZ9102.chr12	13152576	13152577
		arahy.YZ9102.chr12	17819275	17819277
		arahy.YZ9102.chr12	19424537	19424538
		arahy.YZ9102.chr12	19595001	19595005
		arahy.YZ9102.chr12	23637591	23637599
		arahy.YZ9102.chr12	23868563	23869083
		arahy.YZ9102.chr12	119800862	119800863
#13-1-21-2-29	8	arahy.YZ9102.chr12	5021984	5021985
		arahy.YZ9102.chr12	13152576	13152577
		arahy.YZ9102.chr12	17819275	17819277
		arahy.YZ9102.chr12	19424537	19424538
		arahy.YZ9102.chr12	19595001	19595005
		arahy.YZ9102.chr12	23637591	23637599
		arahy.YZ9102.chr12	23868563	23869083

		arahy.YZ9102.chr12	119800862	119800863
#13-1-15-1-4	6	arahy.YZ9102.chr12	5021984	5021985
		arahy.YZ9102.chr12	13152576	13152577
		arahy.YZ9102.chr12	17819275	17819277
		arahy.YZ9102.chr12	19424537	19424538
		arahy.YZ9102.chr12	23637591	23637599
		arahy.YZ9102.chr12	23868563	23869083
#3-1-26-2-33	2	arahy.YZ9102.chr09	119761596	119762045
		arahy.YZ9102.chr19	154138895	154138904
#10-1-23-2-36	2	arahy.YZ9102.chr07	34960798	34960799
		arahy.YZ9102.chr17	2388827	2388828
#10-1-23-1-39	2	arahy.YZ9102.chr07	34960798	34960799
		arahy.YZ9102.chr17	2388827	2388828
#2-1-47-3-41	5	arahy.YZ9102.chr01	45941184	45941362
		arahy.YZ9102.chr01	63810588	63811001
		arahy.YZ9102.chr03	140677641	140677642
		arahy.YZ9102.chr07	74346018	74346236
		arahy.YZ9102.chr15	130716194	130716195
#13-1-16-8-17	No detected			
#20-1-16-5-46	No detected			
#1-3-11-1-30	No detected			
#1-3-2-3-30	No detected			
#1-3-10-9-30	No detected			
#1-3-10-5-30	No detected			
